# Beyond the Scars: An Analysis of Adverse Childhood Experiences and the Interconnections Between Emotion Dysregulation, Dissociation, and Trauma in Patients with Borderline Personality Disorder

**DOI:** 10.3390/brainsci15080889

**Published:** 2025-08-20

**Authors:** Luciana Ciringione, Enrico Perinelli, Francesco Mancini, Elena Prunetti

**Affiliations:** 1 Scuola di Psicoterapia Cognitiva SPC, 37122 Verona, Italy; 2 Department of Psychology and Cognitive Science, University of Trento, 38068 Rovereto, Italy; enrico.perinelli@unitn.it; 3 Associazione di Psicologia Cognitiva APC, Scuola di Psicoterapia Cognitiva SPC, 00185 Rome, Italy; 4 Department of Human Sciences, Guglielmo Marconi University, 00193 Rome, Italy; 5 Centro di Psicoterapia Cognitivo Comportamentale DBT Padova, 35134 Padova, Italy

**Keywords:** borderline personality disorder, PTSD, adverse childhood experiences, dissociation, trauma, emotion dysregulation

## Abstract

**Background/Objectives**: Borderline Personality Disorder (BPD) frequently overlaps with trauma-related conditions, particularly PTSD and Complex PTSD (cPTSD). Adverse Childhood Experiences (ACEs)—especially emotional and sexual abuse—are considered key factors in the development of emotion dysregulation and dissociation. This study investigates the impact of different ACE dimensions on borderline symptomatology, emotion dysregulation, and dissociative symptoms. **Methods**: Eighty-three BPD patients were assessed using standardized self-report questionnaires: CTQ-SF (ACEs), DERS (emotion dysregulation), DES (dissociation), BSL-23 (borderline symptoms), and PDS-3 (post-traumatic symptoms). Analyses included bivariate correlations, Structural Equation Modeling (SEM), and Exploratory Graph Analysis (EGA). **Results**: Emotional abuse significantly predicted borderline symptoms, while sexual abuse predicted dissociation. Emotion dysregulation was strongly associated with both borderline and dissociative symptoms, emerging as a central node in the symptom network. EGA confirmed the clustering of dissociative symptoms with sexual abuse and the centrality of emotion dysregulation across domains. **Conclusions**: Findings support the role of specific ACEs in shaping the clinical expression of BPD. Emotion dysregulation acts as a key transdiagnostic factor linking trauma history to borderline and dissociative features. These results underscore the importance of trauma-informed assessments and interventions, such as DBT and DBT-PTSD, tailored to individual ACE profiles.

## 1. BPD, PTSD, and cPTSD: Diagnoses, Theoretical Models, and Clinical Overlap

Borderline Personality Disorder (BPD), Post-Traumatic Stress Disorder (PTSD), and Complex PTSD (cPTSD) are three interrelated psychopathological conditions. Although they share some symptomatic dimensions, each disorder features distinct characteristics that shape their clinical profiles. Recent research has focused on these specific traits to enhance diagnostic clarity and inform treatment strategies.

BPD [1] is classified among personality disorders, with an estimated prevalence of 1–2% in the general population and substantially higher rates in clinical settings (10–20%), making it one of the most frequently diagnosed personality disorders [2]. According to the categorical model of the DSM-5-TR, BPD is diagnosed by meeting five out of nine main criteria, including: (1) frantic efforts to avoid real or imagined abandonment, (2) unstable and intense interpersonal relationships, (3) identity and self-image disturbances, (4) impulsivity in at least two self-damaging areas, (5) recurrent self-harming behavior and/or suicidal ideation, (6) affective instability due to marked mood reactivity, (7) chronic feelings of emptiness, (8) inappropriate, intense anger or difficulty controlling anger, and (9) transient dissociative symptoms or stress-related paranoid ideation [1]. This diagnostic threshold results in a highly heterogeneous symptom profile, allowing patients with markedly different presentations to fall under the same diagnostic category [3].

The Alternative Model for Personality Disorders (AMPD) in the DSM-5-TR approaches BPD through two main criteria: (A) the level of personality functioning—evaluating impairments in identity, empathy, intimacy, and self-direction—and (B) maladaptive personality traits, such as emotional lability, impulsivity, and hostility, among others. AMPD conceptualizes BPD as resulting from the interplay between an unstable sense of self—marked by self-criticism, emptiness, and dissociation—and problematic interpersonal functioning, often characterized by extreme idealization or devaluation [1]. This dimensional approach helps capture the disorder’s complexity and allows for more tailored therapeutic interventions [4].

Linehan’s biosocial theory [5] conceptualizes BPD as a disorder of emotion dysregulation arising from the interaction between a *biological vulnerability*, characterized by high emotional sensitivity, reactivity, and a slow return to baseline [6], and an *invalidating environment* [7], which reinforces emotional and behavioral difficulties. This model aligns with key components of AMPD Criteria A and B, particularly emotional lability and impulsivity, considering them central to BPD development. However, emerging evidence suggests that the biosocial model may extend beyond BPD, encompassing a broader range of disorders characterized by emotion dysregulation [4].

As previously noted, BPD shares considerable symptom overlap with PTSD [8,9] and with cPTSD [8,9,10]. Indeed, approximately 30% of BPD patients also meet diagnostic criteria for PTSD, and nearly 50% for cPTSD [11,12], as confirmed by recent systematic reviews [13]. This comorbidity may be attributed to shared symptoms across the three disorders, such as emotional hyperreactivity and interpersonal difficulties. Nevertheless, PTSD is typically linked to a single traumatic event, with symptoms including intrusive recollections, avoidance, hypervigilance, and negative alterations in mood and cognition [1]. In contrast, BPD involves chronic dysfunction not necessarily tied to one traumatic episode [14,15]. Zlotnick and colleagues [16] found that among individuals with comorbid BPD and PTSD, childhood trauma was more strongly associated with symptom severity than in those with BPD alone. This suggests that PTSD may amplify clinical complexity and general dysfunction in BPD without altering its core traits.

When PTSD symptoms include disturbances in self-organization (DSO), the diagnosis of Complex PTSD (cPTSD) may apply. DSO encompasses (1) pervasive emotion dysregulation, (2) a profoundly negative self-concept marked by shame and worthlessness, and (3) chronic relational disturbances, such as isolation and difficulty forming stable bonds. The key distinction of cPTSD lies in exposure to prolonged, repeated trauma—particularly interpersonal trauma like emotional, physical, or sexual abuse during childhood. The high prevalence of cPTSD in BPD patients [11] has led some authors to conceptualize cPTSD as a trauma-related subtype of BPD [10,17,18]. However, the specific DSO symptoms are relatively distinct from those of BPD [19,20]. In cPTSD, emotion dysregulation presents as chronic self-regulation difficulties and emotional numbing, rather than the intense mood swings and explosive anger seen in BPD [9]. Furthermore, the negative self-concept in cPTSD is defined by stable, enduring feelings of shame and guilt, contrasting with the unstable and fragmented self-image in BPD. Lastly, while both disorders involve relational difficulties, BPD is marked by intense, alternating patterns of idealization and devaluation to prevent abandonment, whereas cPTSD reflects avoidance rooted in fear of intimacy [19].

### 1.1. Emotion Dysregulation in BPD, PTSD, and cPTSD

Emotion dysregulation lies at the core of BPD. Although also present in PTSD and cPTSD, it manifests in different ways and to varying degrees of severity [9].

In BPD, the interplay between biological and environmental vulnerabilities [5] leads to a chronic inability to modulate intense emotional states. This dysregulation is considered ego-syntonic (i.e., experienced as an integral part of the self) and is often triggered by interpersonal situations perceived as invalidating or threatening [5,8,9]. It reflects a structural instability of identity, a hallmark of BPD [1,15], which appears to be strongly correlated with Adverse Childhood Experiences (ACEs), particularly emotional abuse and emotional neglect [21]. In this regard, a specific analysis by Alafia and Manjula [22] highlighted a significantly higher prevalence of emotional abuse in individuals with BPD compared to clinical control groups, and a substantial association with the development of maladaptive emotion regulation strategies. These maladaptive strategies have been identified as key predictors of negative affective states such as anger and sadness [22].

In PTSD, emotion dysregulation tends to be more specifically linked to traumatic memories and triggers that reactivate the trauma. It is frequently characterized by intense emotional states such as anger, fear, or shame that emerge during flashbacks or in response to trauma-related stimuli [1]. Unlike BPD, dysregulation in PTSD is ego-dystonic (i.e., experienced as alien to the self and closely tied to the traumatic event [8]).

In cPTSD, emotion dysregulation is more persistent and generalized than in PTSD. It involves not only difficulties in modulating negative emotions such as fear and anger, but also a chronic sense of worthlessness and shame that extends to self-perception and interpersonal functioning [23]. It is closely associated with repeated and prolonged interpersonal trauma, which impairs the individual’s capacity to develop adaptive emotional regulation strategies [8,10]. As such, cPTSD is marked by a profound and structural impairment in emotional self-regulation, an element it shares with BPD. Although the overlap between cPTSD and BPD is substantial, Structural Equation Modeling (SEM) studies have revealed that cPTSD symptoms are more closely related to avoidance and relational disconnection, whereas BPD is marked by heightened emotional and relational instability and impulsive behavior [20]. These findings underscore the importance of a precise diagnostic approach that takes into account both the shared features and the differences between the disorders, thereby enhancing the understanding of their interrelations and supporting trauma-focused interventions.

### 1.2. Dissociation in BPD, PTSD, and cPTSD

Dissociation is defined in the DSM-5-TR as a “disruption and/or discontinuity in the normal integration of consciousness, memory, identity, emotion, perception, body representation, motor control, and behavior” [1]. From an evolutionary and pathogenetic perspective, dissociation appears to represent the human organism’s extreme response to intolerable stress, particularly in the context of severe abuse [24].

Since the work of Kernberg [25], dissociation has been conceptualized as an alteration of consciousness and a fragmentation of mental experience, serving an adaptive function in promoting individual survival. This definition has largely remained consistent across different schools of psychotherapy. In the context of BPD, Linehan reconceptualized dissociation as an extreme form of experiential avoidance [26], employed by patients in response to aversive tension states that are perceived as unbearable [5]. The notion of dissociation as experiential avoidance continues to be supported by empirical research on BPD. For instance, a meta-analytic review by Cavicchioli and colleagues [27] confirmed this function across multiple studies.

The link between dissociation and trauma is robust in both Borderline Personality Disorder (BPD) and Dissociative Disorders (DD; [28]), particularly when viewed within a multifactorial etiological framework. A growing body of evidence suggests that ACEs and disorganized attachment are common among individuals with DD, BPD, and cPTSD [29,30,31,32,33,34]. However, patients with BPD tend to exhibit higher levels of dissociation compared to individuals with other psychiatric or personality disorders [35], though not when compared to those with DD or PTSD [36]. This finding may be understood in light of the differing functions that dissociation serves across these disorders.

Specifically, in BPD, dissociation may reflect an acute, situational response to stress [37,38], serving to manage aversive emotional states in the moment, without constituting a structural or stable personality feature [39]. In contrast, in DD, dissociation is a structural element of personality, functioning to protect the individual from traumatic emotions or memories by creating significant compartmentalization within consciousness, identity, and memory [40,41]. Finally, in PTSD, dissociation serves to shield the individual from the traumatic memory. Although it is less chronic than in DD, it is more specifically related to trauma than in BPD [10,42].

In cPTSD, dissociation often manifests as a combination of hyperarousal and hypoarousal, with marked difficulty regulating both positive and negative emotional states. Unlike PTSD, dissociation in cPTSD is closely tied to persistent emotional and relational disorganization, attributed to chronic and prolonged interpersonal trauma [8].

One of the outstanding questions in current research on dissociative symptomatology concerns which specific features of BPD are most strongly associated with dissociation [39]. The present study aims to contribute new evidence on this issue as well.

### 1.3. Adverse Childhood Experiences in BPD, PTSD, and cPTSD

ACEs encompass a wide range of traumatic experiences occurring during childhood, including severe forms of physical, emotional, and sexual abuse, neglect, chronic maltreatment, and invalidating environments [43]. These often cumulative experiences can profoundly impact a child’s psychological, emotional, and neurobiological development, disrupting the ability to regulate emotions, form stable relationships, and develop a coherent and positive sense of self. The effects of ACEs are not limited to childhood but often persist into adulthood, increasing vulnerability to a broad spectrum of psychopathologies, including BPD, PTSD, and cPTSD [10,44]. Their relevance has been extensively studied in both clinical and research settings, consistently identifying ACEs as significant risk factors for long-term emotional and behavioral difficulties.

Patients with BPD are 13 times more likely to report ACEs compared to non-clinical controls and other clinical populations (e.g., mood disorders, psychosis, and other personality disorders [45]), highlighting the central role of ACEs in the development of the disorder [46]. However, not all ACEs exert the same impact. While traumatic events such as physical, emotional, and sexual abuse, neglect, and domestic violence are linked to a greater risk of developing personality disorders in adulthood [44], in BPD, emotional abuse has been found to have the most direct and significant association [22], followed closely by emotional neglect [21]. The evidence regarding the role of sexual abuse is more nuanced. While some studies suggest that sexual abuse is a significant predictor of adult dissociative phenomena—pointing to a specific link between dissociation and sexual trauma (see [47] for a literature review)—more recent findings suggest a more indirect relationship, potentially mediated by other factors such as insecure attachment [46]. This study also aims to shed light on the relationship between sexual abuse, borderline symptomatology, and dissociative and post-traumatic symptoms.

In PTSD, ACEs primarily contribute to the emergence of symptoms related to exposure to single or episodic traumatic events, such as hyperarousal, intrusive traumatic memories, and avoidance behaviors [48]. These symptoms reflect emotional regulation dysfunctions and heightened hypervigilance, which undermine trust in others. While ACEs in PTSD are impactful, their effect tends to be less pervasive than in cPTSD, leading to more circumscribed difficulties rather than widespread and chronic emotion dysregulation [49]. Nonetheless, ACEs remain a critical vulnerability factor, influencing maladaptive emotional responses and psychological functioning, and highlighting the importance of addressing these factors in clinical interventions.

In cPTSD, ACEs—especially when prolonged and repeated—have a profound impact on personality functioning and epistemic trust [49], understood as the ability to trust and learn from social information. Impaired epistemic trust hinders the individual’s capacity to revise self-perceptions and worldviews through positive social interactions, thereby intensifying interpersonal difficulties and the negative self-concept that typify cPTSD. Specifically, the three hallmark Disturbances in Self-Organization (DSO)—emotion dysregulation, negative self-concept, and chronic interpersonal difficulties—emerge from the interplay between multiple, enduring ACEs and impaired personality functioning. Kampling and colleagues [49], in their study on British military veterans, found that those with high exposure to ACEs exhibited more severe cPTSD symptoms, including pronounced DSO, and reported lower levels of perceived social support. These findings underscore the protective role of social support, the absence of which may contribute to the chronic course of the disorder.

Table 1 summarizes the key differences between PTSD, cPTSD, and BPD in relation to the variables examined in this study.

### 1.4. The Present Study

Building on the existing body of literature, the present study aims to address the following research question: How do different dimensions of ACEs influence key psychopathological domains, namely emotion dysregulation, dissociation, and borderline symptomatology?

To this end, the research hypotheses to be tested through bivariate correlation analysis and Structural Equation Modeling (SEM) are as follows:

(1) Different dimensions of ACEs are expected to be positively and significantly correlated with levels of post-traumatic symptomatology, dissociative symptomatology, borderline symptoms, and emotion dysregulation.
(2) Levels of dissociative symptoms are hypothesized to be highly correlated with post-traumatic symptomatology, whereas levels of emotion dysregulation are expected to be strongly correlated with borderline symptomatology.
(3) Emotional abuse and emotional neglect are hypothesized to be the ACE dimensions with the greatest impact on emotion dysregulation and borderline symptoms.
(4) Sexual abuse is hypothesized to be a positive and significant predictor of dissociative symptoms, reflecting a specific link between dissociation and sexual trauma in patients with BPD.

Additionally, Explorative Graph Analysis (EGA) will be used to explore the following research questions:

(1) Which psychopathological factors emerge as central nodes within the symptom network of the tested sample?
(2) Are there patterns that diverge from previously validated measurement structures, thereby suggesting novel latent configurations?

## 2. Methods

### 2.1. Participants

A total of 83 patients (76 females, 7 males) were assessed for the present study between 2019 and 2024.

Participants were voluntarily recruited in a CBT-DBT Psychotherapy Center in Italy, based on the following inclusion criteria: Being 18 years of age or older, being a native Italian speaker, having normative cognitive functioning, and having received a clinical diagnosis of BPD through SCID-5-PD interviews and clinical assessments. At the time of the study, 24 participants had been in psychotherapy for less than three months, 13 for about three months, eight for six months, five for nine months, and 27 for more than nine months. Regarding pharmacological treatment, 22 participants were not taking any medication, 13 were on monotherapy-anxiolytics (*n* = 2), antidepressants (*n* = 5), mood stabilizers (*n* = 2), or atypical antipsychotics (*n* = 3), while 42 were following combined pharmacological regimens.

The study was conducted in accordance with the ethical standards of the 1964 Helsinki Declaration and its later amendments. Ethical approval was obtained from the Ethics Committee of the APC-SPC School of Cognitive Psychotherapy in Rome. Written informed consent was obtained from all participants prior to their participation in the study.

### 2.2. Measures

All participants were administered the Raven’s Progressive Matrices [50] to ensure normative cognitive functioning. Subsequently, the following self-report questionnaires were used to assess the constructs relevant to our research questions:

• ***Borderline Symptom List–23*** (BSL-23; [51]): A short version of the Borderline Symptom List (BSL-95), developed to reliably and efficiently assess symptoms typical of Borderline Personality Disorder. The Italian version consists of 18 items rated on a 5-point Likert scale ranging from 0 (“*not at all*”) to 4 (“*very much*”), measuring symptom severity over the past week. The BSL-23 is designed to be highly sensitive to therapeutic change and to effectively discriminate between patients with BPD and those with other psychiatric diagnoses. The scale has shown excellent internal consistency (Cronbach’s α = 0.89).
• ***Childhood Trauma Questionnaire–Short Form*** (CTQ-SF; [52,53]): A retrospective self-report instrument for measuring childhood trauma. It consists of 28 items assessing five specific dimensions of ACEs: emotional abuse (CTQ_emoab, five items, α = 0.79), physical abuse (CTQ_phyab, five items, α = 0.82), sexual abuse (CTQ_sexab, five items, α = 0.91), emotional neglect (CTQ_emoneg, five items, α = 0.90), and physical neglect (CTQ_phyneg, five items, α = 0.67). Items are rated on a 5-point Likert scale ranging from 1 (“*never true*”) to 5 (“*very often true*”). The scale’s convergent validity is supported by moderate correlations with post-traumatic and general psychopathological symptoms [53].
• ***Difficulties in Emotion Regulation Scale*** (DERS; [21,54]): A self-report questionnaire designed to assess difficulties in emotion regulation. It includes 36 items divided into six subscales that assess clinically relevant aspects of emotion dysregulation: Non-acceptance of emotional responses (DERS_non, six items, α = 0.90), difficulties engaging in goal-directed behavior (DERS_go, five items, α = 0.80), impulse control difficulties (DERS_im, six items, α = 0.90), lack of emotional awareness (DERS_aw, six items, α = 0.85), limited access to emotion regulation strategies (DERS_st, eight items, α = 0.91), and lack of emotional clarity (DERS_cl, five items, α = 0.86). Items are rated on a 5-point Likert scale, ranging from 1 (“*never*”) to 5 (“*always*”).
• ***Dissociative Experiences Scale*** (DES [55,56]): A self-report instrument composed of 28 items designed to measure the frequency of dissociative experiences in clinical and non-clinical populations. Participants rate each item on a visual analog scale from 0% to 100%. The DES assesses three primary dissociative dimensions: absorption and imaginative involvement (DES_assco, nine items, α = 0.84), dissociative amnesia and behavioral lapses (DES_adiss, eight items, α = 0.81), and depersonalization/derealization (DES_depder, six items, α = 0.88). The DES can be used to detect both non-pathological and pathological dissociative symptoms.
• ***Posttraumatic Stress Diagnostic Scale 3*** (PDS-3; [57]): A 24-item self-report measure (PDS_tot, α = 0.89) that assesses the severity of PTSD symptoms over the past month, based on DSM-IV criteria. It evaluates symptom clusters such as intrusive thoughts, avoidance behaviors, and hyperarousal. According to the literature, the PDS-3 is a psychometrically sound instrument for PTSD screening under DSM-IV-R criteria. It is particularly recommended in large-scale traumatic events such as natural disasters or mass terrorism.

### 2.3. Data Analysis

Data analysis was conducted using the open-source statistical software R (version 4.4.2).

Following an initial exploration of descriptive statistics for the sample, bivariate correlations among measured variables were computed using Pearson’s correlation coefficient. In particular, the corrplot package (Version 0.95) was employed to intuitively visualize correlations between questionnaire scores (CTQ, DERS, BSL-23, DES, and PDS), including their subscales, through a correlogram.

For the analysis using SEM [58], the lavaan package (Version 0.6-19) [59] was utilized to test our theory-driven hypotheses derived from the biosocial model of BPD. The following measurement models were specified.

For borderline symptomatology (BSL), three parcels (each composed of six items) were created.

For emotion dysregulation (DERS), a facet-representative approach was used. The latent variable was composed by six observed variables, each representing the composite score (i.e., the average of their respective items) of the six dimensions of DERS: (1) internal state awareness (awareness subscale, DERS_aw), (2) acceptance of internal states (non-acceptance subscale, DERS_non), (3) understanding of emotional responses (clarity subscale, DERS_cl), (4) ability to engage in goal-directed behavior (goals subscale, DERS_go), (5) impulsivity (impulse subscale, DERS_im), and (6) access to effective strategies (strategies subscale, DERS_st).

The same facet-representative approach was applied to dissociative symptomatology (DES), which includes three specific dimensions: (1) absorption and imaginative involvement (DES_assco), (2) dissociative amnesia and behavioral lapses (DES_adiss), and (3) depersonalization and derealization (DES_depder).

The ACEs (via the CTQ) were instead measured by five distinct observed variables, which were computed as the mean of items from each subscale: (1) physical abuse (CTQ_phyab), (2) emotional abuse (CTQ_emoab), (3) sexual abuse (CTQ_sexab), (4) physical neglect (CTQ_phyneg), and (5) emotional neglect (CTQ_emoneg).

Once the measurement models for each construct were defined, the structural model was specified by regressing the latent psychopathology variables (i.e., borderline symptoms, dissociative symptoms, and emotion dysregulation) on the observed ACE variables (i.e., emotional abuse, physical abuse, sexual abuse, emotional neglect, physical neglect). Model fit was evaluated using chi-square statistics (a non-significant result at α = 0.05, i.e., *p* > 0.05, indicates good fit) and the following indices: Comparative Fit Index (CFI; values > 0.90), Tucker-Lewis Index (TLI; values > 0.90), Root Mean Square Error of Approximation (RMSEA; values < 0.06), and Standardized Root Mean Square Residual (SRMR; values < 0.08).

Finally, Exploratory Graph Analysis (EGA) was used to obtain additional insights into the latent factorial structure of the observed variables. Unlike SEM, which tests a predefined structure (i.e., confirmatory or theory-driven approach), EGA identifies factor structures with a data-driven approach, identifying central nodes and interconnections without pre-imposed structures, and, using methods from network science [60]. EGA involves three steps: (1) Estimation of the Correlation Matrix to assess linear relationships among variables; (2) Application of the GLASSO algorithm to build a sparse partial correlation network, highlighting only the strongest associations; and (3) Community Detection and Factor Estimation to cluster items into groups representing latent constructs with strong internal associations. The EGAnet package (Version 2.3.0) in R was used for this analysis.

## 3. Results

Table 2 presents the descriptive statistics for the variables considered in the current sample.

**Figure 1:**
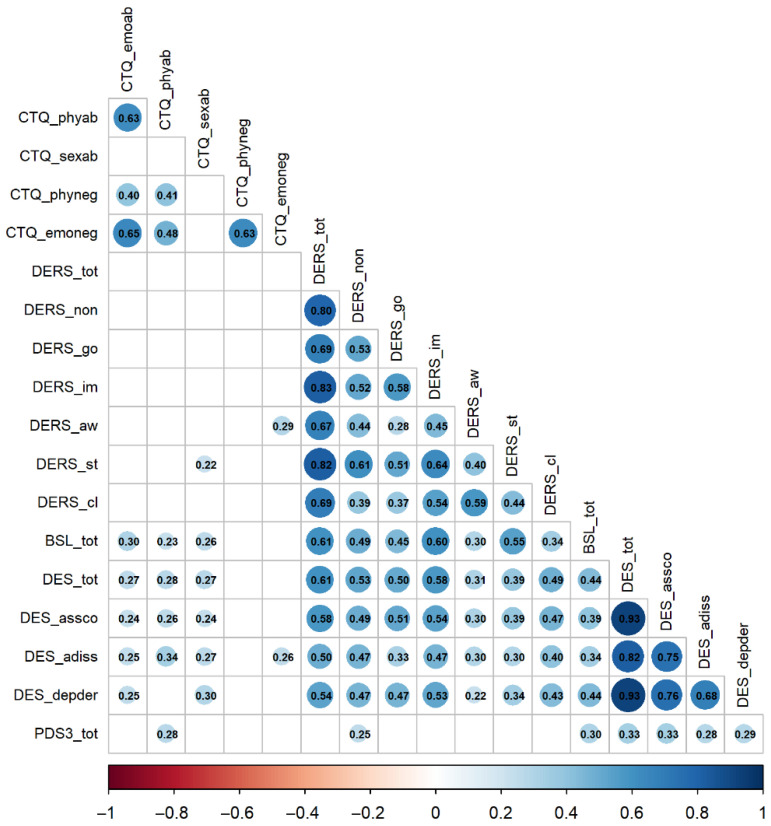
Spearman correlations between study variables. Note. Blank cells represent non-significant correlations at α = 0.05; *p* values are corrected using the Benjamini and Hochberg (BH) procedure. The full matrix is reported in Appendix A, Table A1. BSL: Borderline Symptom List; CTQ: Childhood Trauma Questionnaire; DERS: Difficulties in Emotion Regulation Scale; DES: Dissociative Experiences Scale; PDS3: Posttraumatic Stress Diagnostic Scale.

The correlogram in Figure 1 (see Table A1 in Appendix A for the full correlation matrix) displays the correlations among the various psychopathological variables examined in this study. Bivariate relationships between variables were assessed using Spearman correlations with the Benjamini and Hochberg (BH) procedure for correcting *p*-values; the significance level was set at α = 0.05.

The results of the bivariate correlation analyses suggest the following:

Different dimensions of ACEs show low-to-moderate positive correlations with post-traumatic symptomatology (PDS3_tot, range *ρ* = 0.05–0.28), low-to-moderate correlations with dissociative symptoms (DES_tot, range *ρ =* 0.15–0.28), and low-to-moderate positive correlations with borderline symptomatology (BSL_tot, range *ρ* = −0.05–0.30). Correlations with emotion dysregulation (DERS_tot) were non-significant (range *ρ* = −0.08 to 0.15). These findings are consistent with Hypothesis 1, indicating that the strongest associations are observed with dissociative and borderline symptoms.

Dissociative symptomatology showed a medium correlation with post-traumatic symptoms (PDS3_tot, *ρ* = 0.33, *p* < 0.01), thereby supporting Hypothesis 2. Moreover, dissociative symptoms were also significantly associated with borderline symptomatology (BSL_tot, *ρ* = 0.44, *p* < 0.001), suggesting that dissociation is a salient feature in patients with BPD, often linked to prior traumatic experiences. Levels of emotion dysregulation were strongly correlated with the severity of borderline symptoms (BSL_tot, *ρ* = 0.61, *p* < 0.001), also confirming Hypothesis 2, and with dissociative symptoms (DES_tot, *ρ* = 0.61, *p* < 0.001). These results underscore that emotional difficulties represent a central component in the development and expression of both borderline and dissociative psychopathology.

### 3.1. Structural Equation Models (SEM)

**Figure A1:**
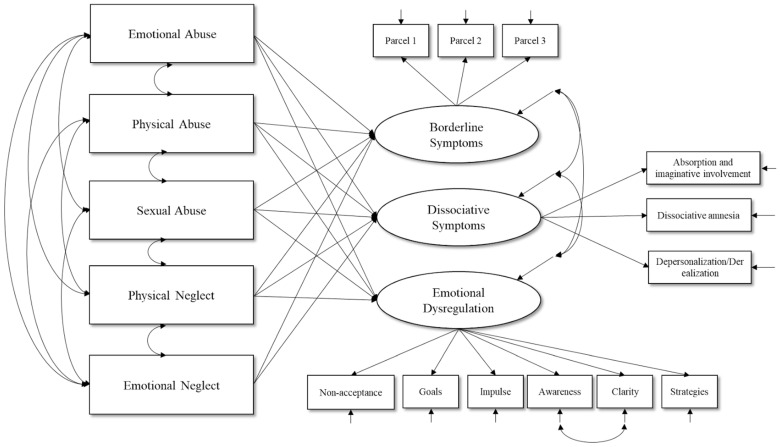
Model 1.

**Figure A2:**
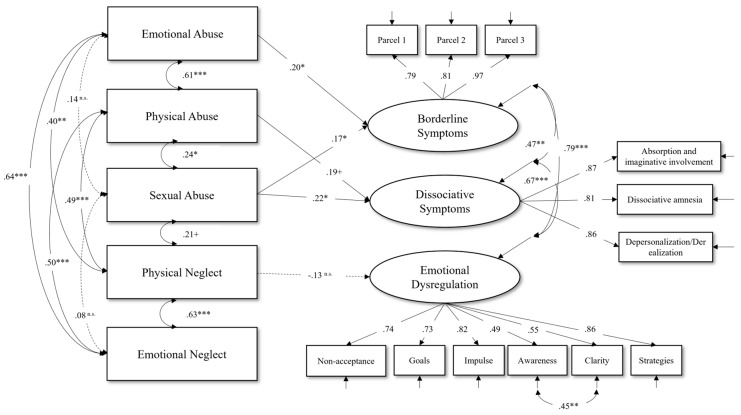
Model 2 with standardized estimates of the free parameters of interest. Note. ^n.s.^ *p* > 0.10. ^+^ *p* ≤ 0.10. * *p* < 0.05. ** *p* < 0.01. *** *p* < 0.001. Solid lines represent significant effects. Dashed lines represent non-significant effects.

The analysis of the structural part of the model was conducted in two phases. In Model 1 (see Figure A1 in Appendix B), it was specified that the three latent outcome variables were regressed on all CTQ subscales. In Model 2 (see Figure A2 in Appendix B), all paths whose standardized estimates had an absolute value less than 0.20 were fixed to zero. Both models were tested using the Maximum Likelihood estimation method, with a total of 78 observations (out of 83 available). In both models, the main fit indices suggest an acceptable fit of the model to the data (Model 1: *χ*^2^ = 136.713, *df* = 95, *p* = 0.003, CFI = 0.945, TLI = 0.921, RMSEA = 0.075, SRMR = 0.060; Model 2: *χ*^2^ = 143.340, *df* = 105, *p* = 0.008, CFI = 0.949, TLI = 0.934, RMSEA = 0.068, SRMR = 0.091).

Partial support was found for Hypothesis 3: emotional abuse (CTQ_emoab) significantly and positively predicted borderline symptomatology (BSL_tot, *β* = .20, *p* < 0.05). However, no significant effects were observed for emotional abuse (CTQ_emoab) on emotion dysregulation, nor for emotional neglect (CTQ_emoneg) on borderline symptomatology (BSL_tot) or emotion dysregulation (DERS_tot).

In support of Hypothesis 4, the effect of sexual abuse (CTQ_sexab) on dissociative symptomatology (DES_tot) was positive and statistically significant (*β* = .22, *p* < 0.05). Additional interesting results from the SEM analysis include a positive and marginally significant effect of physical abuse (CTQ_phyab) on dissociative symptoms (DES_tot, *β* = .19, *p* < 0.10), and a significant effect of sexual abuse (CTQ_sexab) on borderline symptomatology (BSL_tot, *β* = .17, *p* < 0.05).

### 3.2. Exploratory Graph Analysis (EGA)

**Figure 2:**
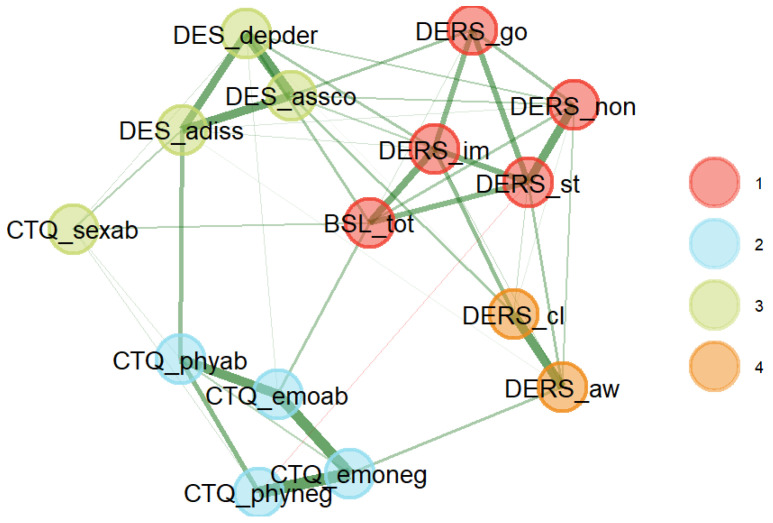
Relationships among ACEs, emotion dysregulation, borderline, dissociative, and post-traumatic symptomatology. Note. BSL: Borderline Symptom List; CTQ: Childhood Trauma Questionnaire; DERS: Difficulties in Emotion Regulation Scale; DES: Dissociative Experiences Scale.

The results of the EGA are reported in Figure 2. The connections between the various nodes are represented by lines of differing thickness. Thicker lines indicate stronger correlations between variables, while thinner or nearly invisible lines represent weaker associations. Variables occupying central positions in the network and showing a high number of connections—so-called “hubs”—may be particularly relevant, as they suggest factors that play a key role in linking different psychopathological nodes. Analyzing the strongest connections and identifying variables that act as bridges between different clusters may provide valuable insights into how various psychopathological domains influence one another.

As illustrated in Figure 2, the nodes are grouped into four distinct clusters, each marked by a different color (red, blue, green, and orange). These clusters suggest the presence of statistically correlated subgroups of variables, which may reflect different psychopathological dimensions or distinct underlying processes.

The green cluster includes variables related to dissociation, such as DES_depder, DES_assco, and DES_adiss, which respectively measure depersonalization and derealization, absorption and imaginative involvement, and dissociative behaviors. These variables are connected by dense and strong links, indicating a high level of correlation among these dissociative phenomena. This result is expected, given that they represent subscales of the same measurement instrument. However, what is particularly noteworthy is the inclusion of the node corresponding to sexual abuse (CTQ_sexab) within the same cluster, highlighting a strong interrelation between dissociative symptomatology and childhood experiences of sexual abuse. This finding further supports Hypothesis 4 of the study.

Borderline symptomatology (BSL_tot) appears to be strongly interconnected with the inability to engage in goal-directed behaviors during emotional distress, the non-acceptance of internal emotional states, and a tendency toward impulsive action—represented by the subscales DERS_go, DERS_non, and DERS_im, respectively. The red cluster specifically emphasizes the centrality of BSL_tot, not only within the red cluster itself but also in relation to other variables, suggesting a possible role of borderline symptoms in linking multiple psychological domains. The centrality of BPD is not surprising, given that the data were collected from a population diagnosed with BPD. Two other subscales of the DERS—namely, clarity (DERS_cl) and impulse (DERS_im)—seem to form a separate cluster (orange cluster), suggesting a unique relationship distinct from the other subscales of the same questionnaire.

The blue cluster, which includes variables such as CTQ_emoneg, CTQ_emoab, and CTQ_phyneg, represents links among various ACE dimensions. This group of variables appears to constitute a separate but meaningful dimension in the network, indicating a strong relationship between early adverse experiences and present manifestations of emotion dysregulation and dissociation. It is worth noting, however, that the variable CTQ_sexab (sexual abuse) shows stronger correlations with dissociative symptomatology than with other childhood adversities measured within the same scale.

Finally, the orange cluster includes awareness of internal emotional states (DERS_aw) and the ability to recognize the nature of emotional responses (DERS_cl) as two distinct dimensions. These two components of emotion dysregulation appear to diverge from the other DERS subscales and from borderline symptomatology (BSL_tot), suggesting a unique pattern within the network structure.

## 4. Discussion

This study has contributed to clarifying the central role of ACEs in the development of BPD. In particular, emotional abuse and emotional neglect are strongly associated with emotion dysregulation, confirming the crucial role of invalidating environments in the development of borderline symptoms, in line with Linehan’s biosocial theory [5]. Emotional vulnerability, exacerbated by such contexts, thus emerges as a key factor in the development of dysregulation and relational difficulties typical of BPD. The data support the hypothesis that emotional abuse and emotional neglect have a stronger correlation with emotion dysregulation than other forms of ACEs in individuals with BPD. Specifically, emotional abuse appears to be more strongly associated with emotion dysregulation than other traumatic experiences, likely due to its direct impact on self-perception and self-esteem [21,22]. Furthermore, both the severity and the type of ACEs experienced significantly influence emotional difficulties, highlighting the importance of clinical interventions focused on improving emotion regulation.

Additionally, the analyses demonstrated that sexual abuse is a significant predictor of dissociative symptoms, suggesting that dissociation may function as a defense mechanism [25] or as a form of experiential avoidance [26,27] in response to severe interpersonal trauma. These findings further support the theoretical models of Ford and Courtois [8] and Herman [10], showing that dissociation acts as a protective mechanism against overwhelming emotions and traumatic memories. Thus, sexual abuse emerges as a central factor in dissociative symptomatology, confirming the original hypothesis and reinforcing the specific and direct link between dissociation and sexual trauma. The SEM analysis strengthens this connection, indicating that dissociation may serve as an adaptive coping strategy in patients with a history of severe interpersonal trauma.

Moreover, the results suggest that emotion dysregulation plays a critical role in the relationship between ACEs and observed symptoms (borderline, dissociative, and post-traumatic), reinforcing the notion that emotion dysregulation constitutes a central node in the symptom network of individuals with BPD. This has direct clinical implications: interventions that enhance emotional awareness, acceptance, and clarity may help to break down the transdiagnostic role of dysregulation, which connects trauma history with current symptomatology. Clinicians should consider individualized assessments of specific emotion regulation deficits, as our findings identified emotional awareness and clarity as distinct dimensions within the network. These facets might require focused interventions to improve patients’ reflective capacities and emotional self-understanding.

The subsequent EGA allowed for a more exploratory investigation of the interconnections among the various domains under study. Findings highlighted emotion dysregulation as a central node in the symptom network, serving as a bridge between ACEs, dissociation, and borderline symptoms. Clinically, this suggests that early identification of dysregulation patterns may offer an effective entry point for intervention, potentially mitigating the cascading effects on dissociative experiences and borderline symptomatology. Although dissociation is prominent in individuals with a history of severe ACEs, it appears to be less central than emotion dysregulation, supporting the view that it may act as a secondary response to severe interpersonal trauma. Nonetheless, when dissociative symptoms are reported in clinical settings, therapists should be vigilant in assessing for possible histories of sexual abuse, as our network analysis revealed a specific and robust link between these variables.

Regarding cPTSD, the findings are consistent with those reported by Powers and colleagues [20], revealing overlap with BPD in the domains of emotion dysregulation and dissociation. However, key differences emerge in self-related symptoms. In cPTSD, emotion dysregulation is chronic and accompanied by persistent emotional numbing and a stable sense of worthlessness or shame. In contrast, BPD is characterized by intense emotional lability, extreme anger, and an unstable and fragmented sense of self [9,19]. These distinctions reflect different origins and adaptive mechanisms in response to trauma: cPTSD is typically associated with prolonged traumatic experiences that compromise the development of adaptive regulation strategies, whereas BPD is marked by a more reactive and impulsive functioning style. These findings underline the importance of accurate diagnostic differentiation, which is essential for developing therapeutic interventions that address the specific features of each disorder.

The results underscore the importance of systematically assessing the presence of ACEs in clinical settings, especially in patients with BPD. Particular attention should be paid to emotional and sexual abuse, in order to identify the most relevant domains of vulnerability and inform successful treatment planning. A detailed clinical exploration of trauma history, integrated with symptom-specific assessments of dissociation and emotion dysregulation, may help clinicians tailor interventions more precisely to individual patients’ needs. The evidence supporting emotion dysregulation as a central node across all symptom domains suggests that therapeutic approaches targeting emotion regulation capacities may be essential components of treatment across diverse clinical profiles. In cases of comorbidity between cPTSD and BPD, treatments should jointly address emotion regulation and trauma processing. Furthermore, the diagnostic distinctions between PTSD, cPTSD, and BPD revealed through EGA and SEM analyses can assist clinicians in making more accurate diagnoses and in developing personalized interventions. For example, patients with marked dissociation and avoidance symptoms may benefit more from trauma-focused interventions, whereas those with BPD may require prioritized interventions targeting emotional regulation and relational instability.

Despite the promising results, the study presents several limitations. First, the relatively small sample size may limit the generalizability of findings to broader clinical populations. Moreover, at the time of assessment, patients were at different stages of psychotherapy and a significant part of the sample was under pharmacological treatment. In addition, only a portion of the sample met diagnostic criteria for PTSD, limiting the robustness of SEM and EGA analyses in exploring PTSD-specific aspects. Additionally, the PTSD assessment tool was based on DSM-IV criteria, and future research may benefit from using updated measures that also capture the DSO components characteristic of cPTSD. A limitation also concerns missing data on the PDS-3, as only participants reporting a specific traumatic event could complete the questionnaire. Moreover, being a self-report measure, some underreporting of traumatic experiences cannot be excluded. Given that this is a cross-sectional study, caution is warranted in generalizing results and in avoiding any causal inference between ACEs and psychopathological symptoms. Furthermore, the cross-sectional nature of the study does not allow for evaluation of the effectiveness of DBT, which the majority of participants had received. Finally, the retrospective assessment of ACEs may be influenced by memory biases, potentially affecting the reconstruction of traumatic experiences.

Future studies would benefit from adopting a longitudinal methodology to further examine the role of emotion regulation as a mediator in the relationship between ACEs and borderline symptoms. Additionally, it would be valuable to explore individual differences in response to emotion regulation-based therapeutic interventions, in order to develop increasingly personalized and evidence-based treatment strategies. Lastly, further research could investigate the interactions between ACEs and other biological and social risk factors in the etiology of BPD.

## 5. Conclusions

This study has contributed to clarifying the central role of ACEs in the development of BPD, highlighting how specific types of ACEs—namely emotional and sexual abuse—are significantly associated with emotion dysregulation and dissociation. The results reinforce the importance of considering ACEs not only as a foundational element in understanding BPD, but also as a critical factor in explaining its symptomatic complexity, which is often associated with comorbidity with PTSD and cPTSD.

The analyses conducted using SEM and EGA demonstrated that ACE dimensions exert a diversified impact on emotion dysregulation, borderline symptoms, and dissociative symptoms in a sample of patients with BPD and trauma. This suggests that difficulties in emotion regulation are not merely a consequence of trauma but represent a central component in the psychopathological network of BPD, linking past trauma to the current manifestation of symptoms.

Another important contribution of this study lies in the differentiation among PTSD, cPTSD, and BPD. While these conditions share symptoms such as emotional hyperreactivity and dissociation, they differ in the origin and nature of those symptoms. The findings emphasize that, whereas cPTSD is more directly associated with chronic and repeated trauma, BPD presents with chronic, ego-syntonic emotion dysregulation that is often unrelated to a single traumatic event.

These results have important implications for clinical practice. Clinicians should systematically assess ACEs—especially emotional and sexual abuse—when working with patients presenting with emotion dysregulation and dissociative symptoms. The identification of specific dysregulation patterns, such as deficits in emotional awareness and clarity, can guide the selection of targeted interventions aimed at enhancing emotional self-regulation. Furthermore, the observed links between dissociation and sexual trauma suggest the need for thorough trauma assessment whenever dissociative symptoms emerge in therapy. Personalized treatment planning, informed by these nuanced diagnostic distinctions, can thus improve therapeutic precision and effectiveness.

## Figures and Tables

**Table 1:** Key differences in emotion dysregulation, dissociation, and Adverse Childhood Experiences among PTSD, cPTSD, and BPD diagnostic frameworks.

Characteristics	BPD	PTSD	cPTSD
Emotion Dysregulation	High and chronic. Includes intense impulses and difficulty managing negative emotions. Ego-syntonic	Present, but specifically related to trauma memories. Ego-dystonic	Persistent and generalized. Associated with a negative self-image and relational difficulties
Dissociation	Frequent, especially in response to stress. May manifest as depersonalization or derealization	Present, linked to specific traumatic events. Typically episodic	More severe and persistent than in PTSD, associated with chronic trauma. Includes derealization and depersonalization
Adverse Childhood Experiences (ACEs)	Very common, particularly emotional abuse, neglect, and relational invalidation	Not necessarily present; often associated with isolated or later-life trauma	Strongly associated with prolonged and repeated ACEs, especially chronic abuse and neglect

**Table 2:** Descriptive statistics of the study variables.

Variable	*n*	*M*	*SD*	Median	Min	Max	Skew	Kurtosis
BSL_tot	82	2	0.79	2.14	0.22	3.89	−0.15	−0.32
BSL_p1	82	1.94	0.82	2	0.17	3.83	0	−0.36
BSL_p2	83	1.99	0.9	2	0.17	4	0	−0.61
BSL_p3	83	2.08	0.89	2.17	0	3.83	−0.31	−0.56
CTQ_emoab	83	13.64	4.96	13	5	24	0.14	−0.68
CTQ_phyab	83	7.71	4.02	6	5	24	1.89	3.66
CTQ_sexab	81	10.12	5.91	8	5	25	0.98	−0.23
CTQ_emoneg	83	15.69	5.06	15	5	25	−0.05	−0.79
CTQ_phyneg	83	7.75	2.8	7	5	20	1.87	4.68
DERS_non	82	19.74	6.5	20	7	30	−0.08	−1.25
DERS_go	81	20	3.66	20	10	25	−0.67	−0.06
DERS_im	82	19.3	5.81	19	8	30	0.01	−0.91
DERS_aw	82	18.78	5.5	19	7	30	−0.24	−0.88
DERS_st	82	29.01	7.32	31	13	40	−0.67	−0.49
DERS_cl	82	15.51	4.26	16	7	24	0.13	−0.81
DERS_tot	81	122.56	25.47	125	66	173	−0.31	−0.62
DES_assco	81	40.64	21.63	37.78	0	86.67	0.14	−0.97
DES_adiss	81	14.37	14.66	8.75	0	65	1.48	1.83
DES_depder	81	30.78	26.45	26.67	0	100	0.77	−0.31
DES_tot	81	28.6	19.02	26.71	0.42	82.04	0.74	0.08
PDS3_tot	66	25.79	11.22	25	0	49	0.19	−0.67

Note. BSL: Borderline Symptom List-23; CTQ: Childhood Trauma Questionnaire; DERS: Difficulties in Emotion Regulation Scale; DES: Dissociative Experiences Scale; PDS3: Posttraumatic Stress Diagnostic Scale.

## Data Availability

The data supporting this study’s findings are available from the corresponding author, Luciana Ciringione, upon reasonable request. The data are not publicly available due to privacy or ethical restrictions.

## Appendix A

**Table A1:** Spearman’s correlations between study variables.

Variable	1	2	3	4	5	6	7	8	9	10	11	12	13	14	15	16	17	18
1. CTQ_emoab	1																	
2. CTQ_phyab	0.63 ***	1																
3. CTQ_sexab	0.13	0.21	1															
4. CTQ_phyneg	0.4 ***	0.41 ***	0.14	1														
5. CTQ_emoneg	0.65 ***	0.48 ***	0.01	0.63 ***	1													
6. DERS_tot	0.15	0.12	0.16	−0.08	0.07	1												
7. DERS_non	0.1	0.06	0.03	−0.04	−0.02	0.8 ***	1											
8. DERS_go	0.11	0	0.08	−0.11	−0.06	0.69 ***	0.53 ***	1										
9. DERS_im	0.16	0.1	0.09	−0.12	0.03	0.83 ***	0.52 ***	0.58 ***	1									
10. DERS_aw	0.07	0.18	0.03	0.1	0.29 **	0.67 ***	0.44 ***	0.28 *	0.45 ***	1								
11. DERS_st	0.15	0.13	0.22 *	−0.18	0	0.82 ***	0.61 ***	0.51 ***	0.64 ***	0.4 ***	1							
12. DERS_cl	0.02	0.05	0.18	−0.01	0.13	0.69 ***	0.39 ***	0.37 ***	0.54 ***	0.59 ***	0.44 ***	1						
13. BSL_tot	0.3 **	0.23 *	0.26 *	−0.05	0.11	0.61 ***	0.49 ***	0.45 ***	0.6 ***	0.3 **	0.55 ***	0.34 **	1					
14. DES_tot	0.27 *	0.28 *	0.27 *	0.15	0.19	0.61 ***	0.53 ***	0.5 ***	0.58 ***	0.31 **	0.39 ***	0.49 ***	0.44 ***	1				
15. DES_assco	0.24 *	0.26 *	0.24 *	0.1	0.19	0.58 ***	0.49 ***	0.51 ***	0.54 ***	0.3 **	0.39 ***	0.47 ***	0.39 ***	0.93 ***	1			
16. DES_adiss	0.25 *	0.34 **	0.27 *	0.17	0.26 *	0.5 ***	0.47 ***	0.33 **	0.47 ***	0.3 **	0.3 **	0.4 ***	0.34 **	0.82 ***	0.75 ***	1		
17. DES_depder	0.25 *	0.19	0.3 **	0.16	0.12	0.54 ***	0.47 ***	0.47 ***	0.53 ***	0.22 *	0.34 **	0.43 ***	0.44 ***	0.93 ***	0.76 ***	0.68 ***	1	
18. PDS3_tot	0.21	0.28 *	0.23	0.05	0.09	0.18	0.25 *	0.14	0.12	0.05	0.06	0.19	0.3 *	0.33 **	0.33 **	0.28 *	0.29 *	1

Note. Spearman’s rho correlations are displayed and *p* values are corrected using the Benjamini and Hochberg (BH) procedure to control for the False Discovery Rate (FDR). * *p* < 0.05. ** *p* < 0.01. *** *p* < 0.001.
